# Genome Sequence of the Parainfluenza Virus 5 Strain That Persistently Infects AGS Cells

**DOI:** 10.1128/genomeA.00653-16

**Published:** 2016-07-21

**Authors:** Elizabeth Wignall-Fleming, Dan F. Young, Steve Goodbourn, Andrew J. Davison, Richard E. Randall

**Affiliations:** a MRC – University of Glasgow Centre for Virus Research, Glasgow, United Kingdom; b Centre for Biomolecular Sciences, School of Biology, University of St. Andrews, St. Andrews, United Kingdom; c Division of Basic Medical Sciences, St. George’s, University of London, London, United Kingdom

## Abstract

We have sequenced the parainfluenza virus 5 strain that persistently infects the commonly used AGS human cell line without causing cytopathology. This virus is most closely related to human strains, indicating that it may have originated from biopsy material or from laboratory contamination during generation of the cell line.

## GENOME ANNOUNCEMENT

Parainfluenza virus 5 (PIV5; genus *Rubulavirus*, subfamily *Paramyxovirinae*, family *Paramyxoviridae* [1]) possesses a single-stranded, negative-sense RNA genome containing seven genes that encode eight proteins (2). This virus has been isolated from numerous species, including humans, monkeys, pigs, cattle, and dogs (3). PIV5 is not known to cause acute disease in humans but has been associated with kennel cough in dogs and acute respiratory symptoms in pigs and calves. It can also cause unrecognized persistent infections of tissue culture cells and is likely to establish persistent infections *in vivo* (4).

The AGS cell line was derived from a human gastric adenocarcinoma (5) and has been used commonly in biomedical research, but it is persistently infected with a strain of PIV5 (4). To determine the genetic characteristics of this strain (PIV5-AGS), RNA was isolated by TRIzol (Invitrogen) extraction of AGS cells (ATCC CRL-1739) and of A549 cells (ATCC CCL-185) infected with the strain isolated from AGS cells. Libraries were prepared using a TruSeq stranded mRNA kit (Illumina) and sequenced using a MiSeq platform (Illumina). The data, which consisted of 2,973,486 and 2,874,556 paired-end reads (301 nucleotides [nt]) for virus from AGS cells and PIV5-AGS-infected A549 cells, respectively, were aligned initially to the PIV5-W3 genome sequence (accession no. JQ743318) (6) using Bowtie2 version 2.2.8 (7), and the alignments were visualized using Tablet version 1.13.08.05 (8). Final corrections to the consensus sequences were then made on the basis of fresh alignments. In each case, examination of the RNA strands from which reads were generated revealed that genomic RNA had copurified with viral mRNA, presumably as a result of intermolecular hybridization, and thus it was possible to determine the genome sequences (15,346 nt). The percentages of the aligned reads were 3.11 and 1.94 for virus from AGS cells and PIV5-AGS-infected A549 cells, respectively. The consensus sequences were almost identical, differing only in the nucleotides at positions 6910 and 7445, which were polymorphic, consisting of mixtures of G and A residues in both genomes. This observation demonstrated that the virus in persistently infected cells is not defective; the possibility of defectiveness had been suggested previously from the observation that AGS cells do not exhibit viral cytopathology (4).

As in several other PIV5 strains (6), the SH gene is unlikely to be functional in PIV5-AGS, because it contains a 1-nt deletion. This deletion is compensated by a 1-nt insertion in the noncoding region between the HN and L genes, the genome thereby conforming to the rule of six (9). Phylogenetic analysis of the PIV5-AGS sequence in comparison with the sequences of 17 other strains available from GenBank showed that PIV5-AGS is most closely related to human strains. Although this finding does not explain the origin of PIV5 in the AGS cell line, it suggests that infection from a human was involved, either because virus was present in the biopsy material or because the cell line was contaminated in the laboratory during its generation.

### Nucleotide sequence accession number.

The PIV5-AGS genome sequence present in persistently infected AGS cells has been deposited in GenBank under the accession no. KX060176.
